# MicroRNAs Profiling Identifies miR-125a and Its Target Gene Wnt2 in Skins of Different Haired Rabbits

**DOI:** 10.3389/fgene.2018.00628

**Published:** 2018-12-10

**Authors:** Yang Chen, Bohao Zhao, Ming Liu, Jingyi Wang, Xiaoqing Qiu, Cigen Zhu, Xinsheng Wu

**Affiliations:** ^1^College of Animal Science and Technology, Yangzhou University, Yangzhou, China; ^2^Jinling Rabbit Farm, Nanjing, China

**Keywords:** rabbit, microRNAs, skin, hair length, miR-125a, Wnt2

## Abstract

MicroRNAs (miRNAs) play critical roles in the control of skin and hair follicle development, epidermal homeostasis and pigmentation. However, the roles of miRNAs in the skins of rabbits with different hair types are unclear. In this study, we profiled miRNAs in the skins of long and short haired rabbits by Illumina deep sequencing. The dataset was compared with known mammalian miRNAs in miRBase 21.0. In total, 118 miRNAs were found to be differentially expressed between the two different rabbit types, of which 94 were upregulated, and 24 were downregulated in the skin of short haired vs. long haired rabbits. The expression levels of five randomly selected miRNAs detected by quantitative real-time PCR indicated that the expression patterns were consistent with Illumina sequencing results. What’s more, bioinformatics analysis showed that miR-125a might target Wnt2, an important modulator for hair follicle development. To test whether Wnt2 is a target of miR-125a, luciferase reporter vector (pMir-report-Wnt2-3′-UTR-WT) and its substitution mutant (pMir-report-Wnt2-3′-UTR-MUT) were constructed. Co-transfection and reporter enzyme assays showed that compared with control (miR-125a NC transfection), miR-125a mimics transfection significantly inhibited the reporter luciferase activities expressed by pMir-report-Wnt2-3′-UTR-WT, while transfection of miR-125a inhibitors increased reporter enzyme activities. RT-PCR and Simple Western analysis found that Wnt2 mRNA and protein levels were induced or repressed by miR-125a overexpression or inhibition, respectively. Moreover, the mRNA expression levels of genes in Wnt signaling pathway, such as CTNNB1, LEF-1, PPARD and TGFB1, were also significantly changed (*P* < 0.05), consistent with Wnt2. It indicated that the regulation of Wnt2 expression by miRNAs may depend on the transcriptional degradation. The results will help to further understand the role of miRNAs in hair follicle development and the genetic mechanism underlying hair length phenotype.

## Introduction

MicroRNAs (miRNAs) are an emerging class of regulators that control post-transcriptional processes and could be potential drug targets as well as potential sites of phenotypic regulation (Lewis et al., 2003; Alvarez-Garcia and Miska, 2005). Some miRNAs have been found to play critical roles in cell differentiation and the proliferation of skin and hair follicles and their target genes play important roles in the periodic growth of hair follicles (Andl et al., 2006; Yi et al., 2006). High throughput sequencing provides a rapid and accurate method for the identification of miRNAs.

Rabbits are economically important fur animals and are used as a model organism for the study of molecular genetics. Although, rabbit miRNAs have been identified by small RNA sequence reads with SOLiD and Illumina platforms (Li et al., 2011), currently, only 12 entries representing hairpin precursor miRNAs and 21 entries for mature miRNA products in rabbits have been identified and deposited in the public miRNA database miRBase. This number is far less than for *Mus musculus* (1,193 precursors, 1,920 mature), *Rattus norvegicus* (495 precursors, 807 mature) and *Homo sapiens* (1,881 precursors, 2,603 mature). Given the identification of the roles of miRNAs in hair follicle development, identifying differentially expressed miRNAs is a key step in investigating the function of miRNAs in rabbit skin.

It is generally known that miRNA/mRNA regulatory networks are involved in the control of skin and hair follicle development, epidermal homeostasis and pigmentation (Botchkareva, 2012). Postnatal hair growth inhibition is due to the aberrant expression of miR-125b in the outer root sheath, which induces a hyper-thickened epidermis and enlarged sebaceous glands (Zhang et al., 2011). MiR-203 is a molecular switch that depends on p63 to promote epidermal differentiation by restricting proliferative potential and inducing cell-cycle exit. Induction of miR-203 in the skin occurs concomitantly with stratification and differentiation (Yi et al., 2008; Wei et al., 2010). MiR-25 not only plays an important role in the regulation of genes linked to coat color, but also in the process of skin and hair development (Zhu et al., 2010). In addition, miRNAs are involved in the regulation of skin and hair development related signaling pathways and factors, such as the Wnt, Notch and Shh signaling pathway (Ryan et al., 2006; Yu et al., 2008), as well as transforming growth factor beta(TGF-β) (Ahmed et al., 2011; Liu X.J. et al., 2013). However, the molecular mechanism underlying the effects of miRNAs in rabbit skin and hair follicle development remains unclear.

Hair length in rabbits is a very important economic trait, which is also crucial in evaluating wool yield and quality. The hair length of Angora rabbits, at approximately 5∼12 cm, and Rex rabbits, at approximately 1.3∼2.2 cm, is significantly different (Gu and Qin, 2013). At present, there are too few studies on the gene mapping of hair length in rabbits, meaning that candidate genes affecting hair length phenotype are currently unknown. In this study, the hybrid offspring of rabbits from the two different hair types were selected for small RNA sequencing to identify the differentially expressed miRNAs and determine the miRNAs and signaling pathways that are involved in hair follicle development. As we all know, Wnt/β-catenin signaling was a classic pathway in initiation and maintenance of primary hair follicle placodes (Zhang et al., 2009). Wnt2 in Wnt signaling pathway played an important role in hair follicle morphogenesis to regulate hair length (Nie et al., 2018). Further, the targeting of Wnt2 by miR-125a, a key differentially expressed miRNA, was identified using a luciferase reporter assay and RT-PCR. It was demonstrated that miR-125a was significantly downregulated in long-haired rabbits. And miR-125a significantly inhibited Wnt2 mRNA and protein expression and reduced the luciferase activity of Wnt2-3′-UTR. The results will help to further understand the role of miRNAs in hair follicle development and the genetic mechanisms behind hair length phenotype.

## Materials and Methods

This study was carried out in accordance with the recommendations of Animal Care and Use Committee at Yangzhou University. The protocol was approved by the Animal Care and Use Committee at Yangzhou University.

### Tissue Collection and RNA Extraction

The Wanxi Angora rabbits and Rex rabbits were provided by the Anhui Academy of Agricultural Sciences, Hefei, Anhui, China. Three healthy long-haired rabbits (8 months old) and three short haired rabbits (8 months old) were selected in November. The hair length of both types of rabbits was measured, with the long-haired rabbits having significantly longer hair than the short haired rabbits. The information on the selected animals is shown in Table 1. A 1 cm^2^ skin tissue sample was obtained from the back, placed immediately in liquid nitrogen, and preserved at -80°C until use. The iodine solution was smeared on the resultant lesion to prevent bacterial infection. Total RNA was extracted using the mirVana^TM^ miRNA Isolation Kit (Austin TX, United States) according to the manufacturer’s instructions. The total RNA quantity and purity were analyzed using a Bioanalyzer 2100 (Agilent, CA, United States) and RNA 6000 Nano LabChip Kit (Agilent, CA, United States) with RIN number > 7.0.

**Table 1:** Information on the selected rabbits.

No.	Marker	Body weight(g)	Hair type	Hair length(mm)
L1	2665	2534	Long	79.1∼113.4
L2	2489	2880	Long	52.1∼92.3
L3	2651	2591	Long	67.8∼93.6
S1	2627	2325	Short	25.7∼34.1
S2	2799	2368	Short	25.8∼35.1
S3	2641	2262	Short	26.8∼36.5

### Small RNA Library Construction and Sequencing

Approximately 1 μg of total RNA was used to prepare a small RNA library according to the protocol included with the TruSeq small RNA sample prep kit (Illumina, San Diego, CA, United States). The small RNA library was constructed using each sample at the same concentration and single-end sequencing (50 nt) was performed on an Illumina Hiseq2500 (Illumina, San Diego, CA, United States) following the vendor’s instructions.

The raw reads were filtered by the Illumina pipeline and then the dataset was further processed to remove adapter dimers, low complexity reads, reads mapping to common RNA families (rRNA, tRNA, snRNA, and snoRNA) and repeats. The clean reads were mapped to the genomes of *Oryctolagus cuniculus* and other mammals using SOAP software to analyze their expression and distribution(Li et al., 2008). Matched sequences were blasted against the NCBI Rfam database and GenBank database to identify and remove rRNA, tRNA, snRNA, scRNA, srpRNA, and snoRNA sequences. The remaining high-quality sequences were subsequently compared to rabbit miRNAs, as well as to other animal miRNAs, in miRBase 21.0. Sequences in the libraries with identical or related sequences (1∼2 nucleotide substitutions permitted) to *Oryctolagus cuniculus* or other mammals (*Homo sapiens, Mus musculus, Sus scrofa, Bos taurus, Ovis aries, Capra hircus, Cricetulus griseus, Equus caballus, Pan troglodytes and Rattus norvegicus*) were identified as conserved miRNAs.

Sequencing reads that did not match any of the known miRNAs were further analyzed to identify novel miRNAs. miRDeep2 and RNAfold software were also used to predict typical secondary structures for the miRNA precursors and remove pseudo pre-miRNAs. For every novel miRNA, false positive rate (FPR) and true positive rate (TPR) were used to determine accuracy using miRDeep2.

### Analysis and Target Gene Prediction of Differential Expressed miRNAs

To compare differentially expressed miRNAs in the long haired (L library) and short haired (S library) rabbits, the expression levels of miRNAs in the two groups were normalized to obtain the expression of transcripts per 1,000,000 (TPM). The formula used was Normalized expression = (actual miRNA count / Total count of clean reads) × 1,000,000. The fold-change and *p*-value were calculated from the normalized expression. Negative binomial distribution in DESeq package was used to analyze differentially expressed genes (Anders and Huber, 2013). The miRNAs were selected based on *P*-values < 0.05 and | log_2_ (fold change)| > 1. The two computational target prediction algorithms (TargetScan and miRanda 3.3a) were used to predict target genes of the differentially expressed miRNAs. Only when the target was identified by both programs, it was considered to be the potential target for a given miRNA. The miRNA-gene network was constructed using Cytoscape software (Cytoscape v3.4.0) to analyze the interactions between miRNAs and genes.

### Validation of Differentially Expressed miRNAs Using qRT-PCR

Some differentially expressed miRNAs were validated using qRT-PCR with SYBR green. The primers for qRT-PCR are listed in Table 2. qRT-PCR was carried out on a QuantStudio^TM^ 5 Real-Time PCR System (Bio-Rad, United States) with SYBR Green PCR Master Mix (TaKaRa, Dalian, China). The reaction mixtures were incubated in a 96-well plate at 95°C for 30 s, followed by 40 cycles of 95°C for 10 s, 60°C for 10 s, and 68°C for 20 s. U6 snRNA was used as the reference gene. All reactions were performed in technical triplicates. Relative quantification of expression was calculated using the 2^-ΔΔCt^ method after the threshold cycle (Ct) and was normalized with the Ct of U6 snRNA.

**Table 2:** Primers used in this study.

miR-name	miR sequences (5′–3′)	Primer sequences (5′–3′)
miR-125a	TCCCTGAGACCCTTTAACCTGT	GTCCCTGAGACCCTTTAACCTGT
miR-23b	ATCACATTGCCAGGGATT	GGAATCACATTGCCAGGG
miR-31	AGGCAAGATGCTGGCATAGCTG	AGGCAAGATGCTGGCATAGCT
miR-17	CAAAGTGCTTACAGTGCAGGTAG	CAAAGTGCTTACAGTGCAGGTAG
miR-20a	TAAAGTGCTTATAGTGCAGGTAG	TAAAGTGCTTATAGTGCAGGTAG
U6 snRNA		CAAGGATGACACGCAAATTCG

### Plasmid Construction and Dual-Luciferase Assay

The miR-125a mimics, miR-125a mimics NC (negative control), miR-125a inhibitors and miR-125a inhibitors NC (negative control) were purchased from Guangzhou RiboBio Co., Ltd. Specific miRNA target sequences were inserted between the *MluI*–*SpeI* restriction sites in the pMir-report vector (AM5795; Promega, Madison, WI, United States). The luciferase reporter vector (pMir-report-Wnt2-3′-UTR-WT, WT) and its substitution mutant (pMir-report-Wnt2-3′-UTR-MUT, MUT) were constructed. Co-transfection and reporter enzyme assays showed that compared with control (miR-125a NC transfection), miR-125a transfection significantly inhibited the reporter luciferase activities expressed by pMir-report-Wnt2-3′-UTR-WT, while transfection of miR-125a inhibitors increased reporter enzyme activities.

Luciferase activity assay was performed using the Dual-Luciferase Reporter Assay System (Promega, Madison, WI) according to the manufacturer’s instructions. RAB-9 cells of 85-90% confluence were seeded in 96-well plates. For Wnt2 3′UTR luciferase reporter assay, 100 ng WT or MUT were co-transfected into RAB-9 cells with 50 ng pMir-report-UTR and 10 ng pRL-TK using Lipofectamine^TM^ 2000 (Invitrogen). Luciferase activity assay was performed 48 h after transfection using the Dual-Luciferase Assay System. Firefly luciferase activity was normalized to the corresponding Renilla luciferase activity. All the experiments were performed three times.

### Simple Western Analysis

The Cell lysis buffer for Western (Beyotime) was mixed with PMSF (with a final concentration of 1 mM) and added to cell samples, which were centrifuged at 10,000*g* for 5 min at 4°C. Protein lysates were obtained and Enhanced BCA Protein Kit (Beyotime) used for the detection of protein concentrations. Then the protein lysates were diluted to 0.5 μg/μL, and Simple Western analysis was performed using the Wes Simple Western (Protein Simple) system according to the manufacturer’s instructions (Harris, 2015).

## Results

### Small RNA Library Construction and Illumina Sequencing

To systematically identify small RNAs and changes in the expression levels of miRNAs in the long and short haired rabbits, we purified and sequenced small RNAs isolated from rabbit skin. First, raw reads were obtained from the L and S libraries. After removing low-quality reads, adaptors, and insufficient tags, clean reads were obtained (Table 3). The miRNA sequencing data have been deposited in GenBank under the bioproject PRJNA395429.

**Table 3:** Summary of reads from raw data to cleaned sequences for all small RNAs.

Sample	Raw reads	Reads trimmed length	Reads trimmed Q20	Reads trimmed N	Clean reads	Clean reads uniq
L1	36870542	13307420	113450	25923	23423749	1441446
L2	27236688	4907640	153708	29677	22145663	1397488
L3	30027550	9505901	99087	22554	20400008	1790815
S1	34343633	3508802	138503	34403	30661925	865290
S2	29123096	4806044	171041	31692	24114319	1867851
S3	32520271	1498835	127125	34076	30860235	985393

**FIGURE 1:**
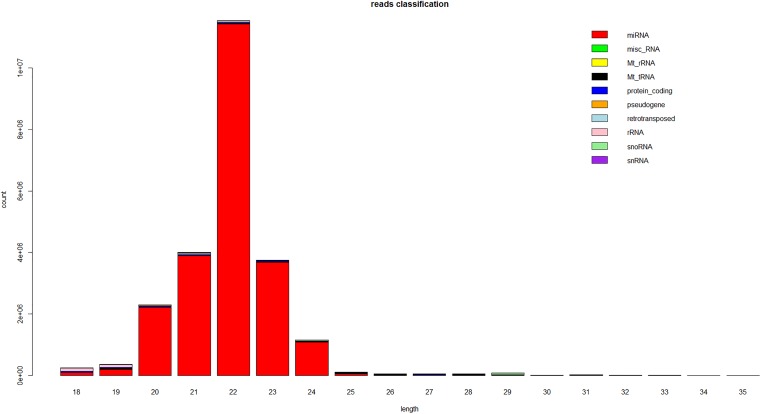
Length distribution of the sequence reads that mapped to the rabbit genome from the small RNA libraries. This is a representative result from one of the six samples.

The size distribution of the small RNAs was similar in both libraries. The lengths of most of the small RNAs were between 18 and 24 nt, with the most common size of the small RNAs being 22 nt, which accounted for 30% ∼ 40% of the small RNAs in the skins of long and short haired rabbits (Figure 1). Subsequently, the small RNAs were classified into several different categories based on their annotations. Sequences were compared with the known noncoding RNAs deposited in the Rfam and NCBI GenBank databases and miscRNAs, rRNAs, snoRNAs and snRNAs were separated from the miRNAs and discarded. Small RNA tags were aligned to repeat-associated RNA to find matched tags in the samples.

### Identification of Conserved miRNAs and Novel miRNA Candidates

**FIGURE 2:**
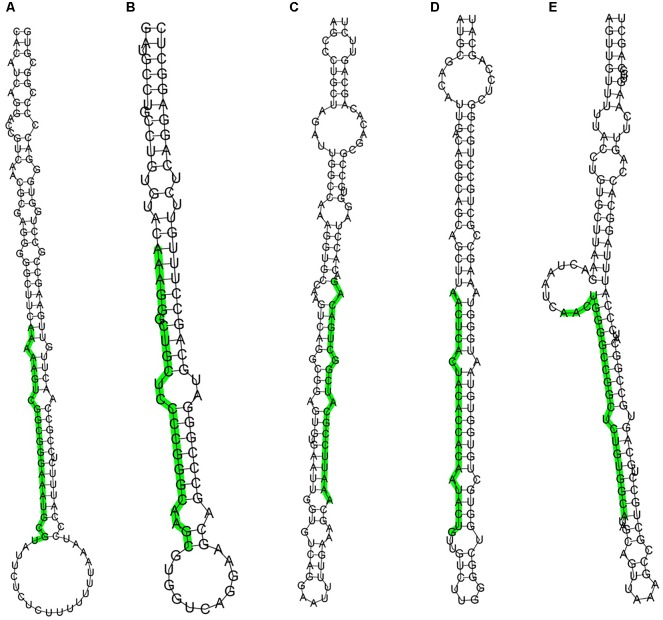
Stem-loop structures of 5 selected novel rabbit miRNAs. The secondary structures were generated from the rabbit genome. The uppercase letters refer to mature sequences and are indicated in green. **A**: GL018776_1023424-1023445, **B**: 13_70851768-70851790, **C**: GL018831_682733-682753, **D**: 9_17061322-17061343, **E**: 13_137090062-137090083.

As there are only 12 entries representing hairpin precursor miRNAs and 21 entries for mature miRNA products from rabbits in the Rfam and Genbank databases, to identify known miRNAs in rabbit skin, the dataset was compared with known mammalian miRNAs (miRNA precursors and mature miRNAs) in miRBase 21.0. We identified 2273, 2094, 2171 conserved miRNAs in the L libraries and 1848, 1660, 1719 in the S libraries. Considering the conservation of mature miRNAs among various species, the sequences of the existing miRNAs in rabbits were aligned and analyzed based on their evolutionary relationships. Sequencing reads that did not match any of the conserved miRNAs were further analyzed to identify novel miRNAs. The rabbit genome was also utilized to identify potential novel miRNAs. In total, 234 miRNA candidates were identified as having a typical miRNA stem-loop secondary structure, which forms the Dicer enzyme cleavage site. The hairpin structures for these novel miRNAs are denoted in Supplementary Table S1. The structures of 5 potential novel miRNA precursors, such as GL018776_1023424-1023445, 13_70851768-70851790, GL018831_682733-682753, 9_17061322-17061343 and 13_137090062-137090083, are shown in Figure 2. The presence of stem-loop hairpin secondary structures of the 5 sequences is similar to the results with the conserved miRNAs.

### Differential Expression of Conserved miRNAs and Validation of miRNA Expression by qRT-PCR

Pairwise comparisons revealed important differentially expressed miRNAs between rabbits of the two different hair types. In total, we identified 118 differentially expressed miRNAs from the L and S libraries (Supplementary Table S2). Of these 118 miRNAs, 94 were down-regulated in the skin of short haired rabbits compared to the skin of long haired rabbits, and 24 miRNAs were up-regulated with a fold change greater than 2, such as mir-31, mir-34c, mir-34b, mir-146a,mir-204 and so on.

**FIGURE 3:**
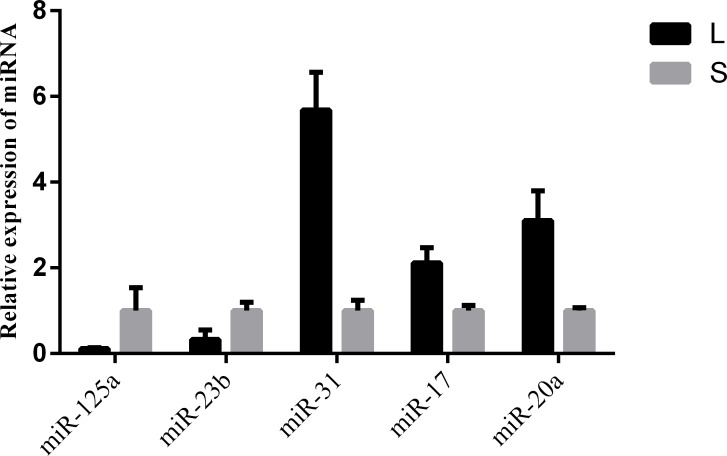
qPCR validation of miRNA expression in skin samples from rabbits with different hair types. L and S indicated L and S libraries, respectively. miR-125a and miR-23b were up-regulated in S libraries, and miR-31, miR-17 and miR-20a were up-regulated in L libraries. The expression pattern of these miRNAs was consistent in miRNA-seq and qRT-PCR experiments.

To validate the miRNA expression level changes and gain insight into the possible roles of miRNAs related in hair follicle function and development, 5 conserved miRNAs (miR-125a, miR-23b, miR-31, miR-17 and miR-20a) were assessed by qRT-PCR. miR-125a and miR-23b were up-regulated in short haired rabbits, and miR-31, miR-17 and miR-20a were up-regulated in long haired rabbits. The expression pattern of these miRNAs was consistent in miRNA-seq and qRT-PCR experiments (Figure 3).

### Network Analysis Between miRNAs and Target Genes

Target Scan V.6.0 was used to predict the gene targets of the differentially expressed miRNAs. As a result, a total of 13,354 annotated mRNA transcripts were predicted as putative targets of the differentially expressed miRNAs. From our analysis, it appeared that one miRNA could target multiple genes, for example, 80 annotated mRNA transcripts were predicted as putative targets for miR-21, which was the most differentially expressed miRNA in rabbits. Moreover, our results indicated that some genes were regulated by more than one miRNA.

**FIGURE 4:**
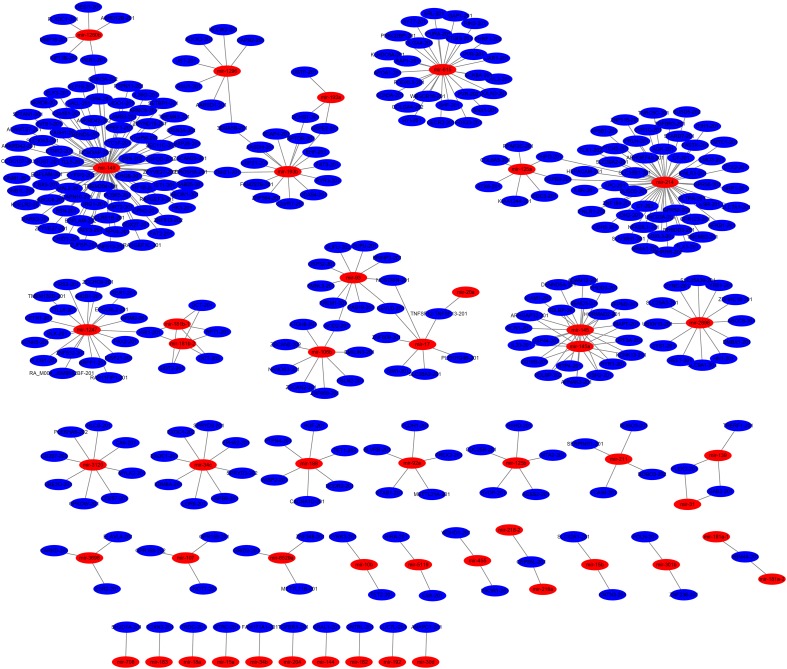
miRNA-gene network between rabbits with different hair types. The miRNA-gene network further revealed core genes and miRNAs involved in the development of skin and hair follicles.

Combined with the RNA sequencing data (deposited in GenBank, PRJNA352481), we constructed an interaction network of miRNAs and target genes using Cytoscape software (Figure 4 and Supplementary Table S3). It revealed miRNAs as core node involved in the development of skin and hair follicles, such as mir-149, mir-214, mir-17, and mir-193b, may play important roles in hair follicle development. The microRNA/mRNA regulatory relationships related hair follicle development were more accurately explored, such as mir-17/SLC20A2-201, mir-145/SSH2-201 and so on.

### Identification of Wnt2 of miR-125a Target Gene

**FIGURE 5:**
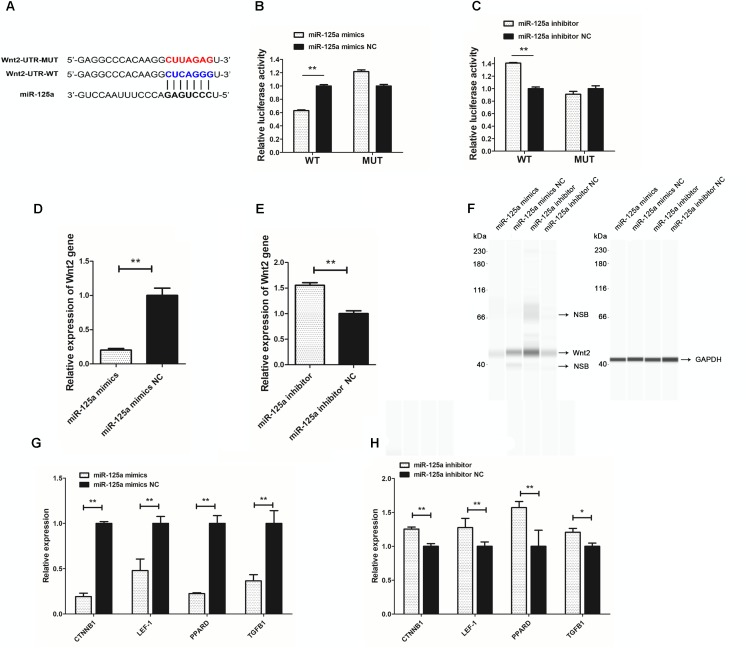
miR-125a directly regulates Wnt2. **(A)** Putative miR-125a-binding sites (red letters) and mutated sites (blue letters) in the 3′-UTR of rabbit Wnt2 in the pMir-report Dual-Luciferase miRNA Target Expression Vector. **(B)** Luciferase activity assays of RAB-9 cells co-transfected with Wnt2 3′-UTR-WT and a miR-125a mimics,Wnt2 3′-UTR-MUT and a miR-125a mimics. **(C)** Luciferase activity assays of RAB-9 cells co-transfected with Wnt2 3′-UTR-WT and a miR-125a inhibitors, Wnt2 3′-UTR-MUT and a miR-125a inhibitors. The activity of the firefly luciferase protein was normalized to Renilla luciferase activity. **(D)** The effect of miR-125a mimics on the endogenous Wnt2 gene expression in RAB-9 cells. **(E)** The effect of miR-125a inhibitors on the endogenous Wnt2 gene expression in RAB-9 cells. **(F)** Simple Western analysis of Wnt2 (40 kDa) expression when miR-125a was over-expressed or inhibited. GAPDH (36 kDa) as the internal control. NSB stood for non-specific binding. **(G)** The effect of miR-125a overexpression on the mRNA expression of CTNNB1, LEF-1, PPARD and TGFB1. **(H)** The effect of miR-125a inhibition on the mRNA expression of CTNNB1, LEF-1, PPARD and TGFB1. ^∗^*P* < 0.05, ^∗∗^*P* < 0.01.

To search for putative direct miR-125a target mRNAs, we used TargetScan, miRanda and RNAhybrid (Li and Zhang, 2016). Among the candidate target genes, it was found Wnt2 was associated with hair follicle development and had putative binding site in its 3′UTR (Figure 5A). miR-125a targeting elements in the Wnt2-3′-UTR are highly conserved in many mammals.

To determine whether miR-125a is able to recognize the Wnt2-3′-UTR, we generated a luciferase reporter DNA construct pMir-report-Wnt2-3′-UTR-WT with a miR-125a putative binding site. When miR-125a mimics was cotransfected with the luciferase reporter vector into RAB-9 cells, luciferase activity was significantly suppressed (Figure 5B). In order to determine the specificity between miR-125a and Wnt2-3′-UTR target site, luciferase reporter was mutated at the target elements in the Wnt2-3′-UTR which are the “seed” match region. Unlike wild-type luciferase reporter, miR-125a expression could not suppress the mutant reporter activity (Figure 5B). When miR-125a inhibitor was cotransfected with the luciferase reporter vector into RAB-9 cells, luciferase activity was significantly increased, and not increased the mutant reporter activity (Figure 5C). These results clearly indicated that miR-125a would directly recognize and bind to the 3′-UTR of Wnt2.

In order to understand the molecular mechanism of miR-125a targeting Wnt2 deeply, we analyzed the effect of miR-125a mimics and inhibitors on Wnt2 mRNA and protein expression. The results showed that expression of Wnt2 could be down-regulated or up-regulated by miR-125a mimics or inhibitors compared with NC transfection (Figures 5D,E). And Wnt2 protein abundance changed significantly after miR-125a overexpression or inhibition, as assessed by Simple Western analysis (Figure 5F). In addition, the mRNA expression levels of genes in Wnt signaling pathway, such as CTNNB1, LEF-1, PPARD, and TGFB1, were also significantly changed (*P* < 0.05), consistent with Wnt2 (Figures 5G,H).

## Discussion

MicroRNAs are important regulators of post-transcriptional gene expression and are a potential target for drugs, as well as physiological sites for phenotypic regulation. Knowledge on the function of microRNAs and their mechanism of action in skin follicle development is not entirely clear. High-throughput sequencing has provided a way to identify microRNAs accurately and rapidly. In this study, we analyzed miRNAs in long and short haired rabbits in order to gain further information about the use of rabbits as animal models in miRNA studies. A positive result will have an obvious influence on identifying skin follicle development-related microRNAs and examining the important role that the target genes play in regulating the periodic growth of hair follicles.

Based on previous studies in humans, mice and other mammals, it is known that hair length is related to the duration of the growth cycle and is regulated by the changes in the hair follicle cycle. There are 3 known phases in this dynamic cycle, namely the growth phase (anagen), cessation phase (catagen) and rest phase (telogen) (Paus and Foitzik, 2004). Transitions between the phases are influenced by interactions between a series of growth signals and inhibitory molecules, which in turn leads to the continuous differentiation and growth of hair follicles. In recent years, many studies have used high-throughput sequencing to identify and predict skin hair follicle development-related microRNAs. Sequencing of skin hair follicle tissues at different phases of growth from *Ovis aries* and *Capra hircus* has identified several miRNAs that are important for the development of the skin and hair follicles, and these miRNAs were predicted to regulate hair follicle growth through regulating target genes in the MAPK and Wnt pathways(Liu Z. et al., 2012; Liu G. et al., 2013; Yuan et al., 2013). The high-throughput sequencing was used to identify seven candidate target genes from hair follicle morphology-related microRNAs in ducks, and these genes were found to be associated with the Wnt/β-catenin, Shh/BMP and Notch pathways. Furthermore, the microRNAs and microRNA families involved in the growth of feather follicles and mammalian hair follicles were also found to be different (Zhang et al., 2013). In the present study, we found that 118 miRNAs were differentially expressed between the two different rabbit types. Some interesting miRNAs were explored in different studies, such as mir-214, mir-149, mir-31, mir-17, mir-34, mir-125a and mir-193b and so on. Those miRNAs were suggested that may play an important role in the development of skin and hair follicles, and act on various signaling pathways to regulate transitions between each phase of the hair follicle cycle and thereby control hair length. However, rabbit hair coats were shaped by complex factors. Using RNA samples prepared from heterogeneous tissues were flawed. So purified cells were carried in confirmatory studies.

The microRNA/mRNA regulatory relationship plays an important role in the regulation of skin and hair follicle development. A previous study showed that miR-31 could negatively regulate Sclerostin and BAMBI elements in the Wnt and BMP signaling pathways, as well as fibroblast growth factor-10 (Fgf10), and the expression of Dlx3 transcription factor and keratin. miR-31 was significantly upregulated during anagen of hair follicles in mice and was shown to promote hair cell differentiation and hair formation (Mardaryev et al., 2010). BMPs are important signaling molecules involved in hair follicle development. miR-21 is expressed in epidermal cells and hair follicle epithelial cells of normal mice and negatively regulates target genes in the BMP signaling pathway (Rendl et al., 2008; Ahmed et al., 2011). We combined the miRNA data with the RNA sequencing data, and obtained the network of miRNA–mRNA network. It revealed some core miRNAs and gene involved in the development of skin and hair follicles, such as SLC30A3/mir-149 and SLC7A1/mir-214. These findings would provide new sights for understanding the molecular mechanisms underlying hair length phenotype. Based on the above analyses and qRT-PCR validation results, we speculate that several miRNAs, such as miR-31, miR-125a, miR-17 and miR-21, are involved in the development of skin and hair follicles in rabbits.

MiRNAs served as crucial post-transcriptional regulators of gene expression by transcriptional degradation of target mRNA or inhibition of protein synthesis (Li et al., 2016; Miao et al., 2017; Shao et al., 2017). In order to reveal the microRNA/mRNA regulatory relationship, we predicted that miR-125a could target Wnt2, belonging to the Wnt genes family. It is well known that activation of Wnt signaling in the skin precedes is required for the localized expression of regulatory genes and initiation of hair follicle placode formation (Andl et al., 2002). According to reports, Wnt2 takes part in regulation of proliferation, differentiation and apoptosis of cells and has previously been linked to the progression of cancer. In addition, Wnt2 likely acts as the secondary Wnt, which is a part of the placode signal and maybe is very essential for HF initiation (Yue et al., 2016). In present, there is no any report involved in miRNA target Wnt2 in follicles development. Our results clearly indicated that miR-125a would directly recognize and bind to the 3′-UTR of Wnt2. Some miRNAs lead to the degradation of target gene mRNA and regulate the expression of target genes. Our results indicated that the regulation of Wnt2 expression by miRNAs may depend on the transcriptional degradation. Moreover, miR-125a was involved in hair follicles development by effecting on the mRNA expression levels of genes in Wnt signaling pathway, such as CTNNB1, LEF-1, PPARD and TGFB1, consistent with Wnt2.

In summary, the candidate miRNAs involved in hair follicle development of rabbits were revealed by Illumina deep sequencing. Further, the targeting of Wnt2 by miR-125a, one of candidate miRNAs, was identified. This study serves as the basis for function of miRNAs in rabbit hair follicle development.

## Author Contributions

YC conducted the analysis and wrote the manuscript. BZ carried out the part of experiments. ML, JW, and XQ contributed to the dataset for this study. CZ and XW designed the study and finalized the manuscript. All authors read and approved the final manuscript.

## Conflict of Interest Statement

The authors declare that the research was conducted in the absence of any commercial or financial relationships that could be construed as a potential conflict of interest.

## Supplementary Material

TABLE S1 Hairpin structures for novel miRNAs. The detailed information of secondary structures of the precursors of the novel miRNAs were showed.

TABLE S2 Differentially expressed miRNAs from the L and S libraries. The expression of the miRNA in two libraries was normalized to get the expression of TPM. The fold-change and p-value were calculated from the normalized expression. The differentially expressed miRNAs were selected based on *P*-values < 0.05 and | log2 (fold change)| > 1.

TABLE S3 miRNAs and target genes for the miRNA-gene network construction. TargetScan and miRanda 3.3a were used to predict target genes of the differentially expressed miRNAs. Only when the target gene was identified by both programs, it was considered to be the potential target gene for the given miRNA.

## References

[B1] Ahmed M. I. Mardaryev A. N. Lewis C. J. Sharov A. A. Botchkareva N. V. (2011). MicroRNA-21 is an important downstream component of BMP signalling in epidermal keratinocytes. *J. Cell Sci.* 124 3399. 10.1242/jcs.086710 21984808 PMC3196856

[B2] Alvarez-Garcia I. Miska E. A. (2005). MicroRNA functions in animal development and human disease. *Development* 132 4653–4662. 16224045 10.1242/dev.02073

[B3] Anders S. Huber W. (2013). *Differential Expression of RNA–Seq Data at the Gene Level – The DESeq Package.* Heidelberg: European Molecular Biology Laboratory, 1–23.

[B4] Andl T. Murchison E. P. Liu F. Zhang Y. H. Yunta-Gonzalez M. Tobias J. W. (2006). The miRNA-processing enzyme dicer is essential for the morphogenesis and maintenance of hair follicles. *Curr. Biol.* 16 1041–1049. 16682203 10.1016/j.cub.2006.04.005 PMC2996092

[B5] Andl T. Reddy S. T. Gaddapara T. Millar S. E. (2002). WNT signals are required for the initiation of hair follicle development. *Dev. Cell* 2 643–653. 12015971 10.1016/s1534-5807(02)00167-3

[B6] Botchkareva N. V. (2012). MicroRNA/mRNA regulatory networks in the control of skin development and regeneration. *Cell Cycle* 11 468–474. 10.4161/cc.11.3.19058 22262186

[B7] Gu Z. L. Qin Y. H. (2013). *Rabbit Production in China.* Beijing: China Agriculture Press.

[B8] Harris V. M. (2015). Protein detection by Simple Western^TM^ analysis. *Methods Mol. Biol.* 1312 465–468. 10.1007/978-1-4939-2694-7_47 26044028

[B9] Lewis B. P. Shih I. H. Jones-Rhoades M. W. Bartel D. P. Burge C. B. (2003). Prediction of mammalian microRNA targets. *Cell* 115 787–798. 14697198 10.1016/s0092-8674(03)01018-3

[B10] Li C. Zhang P. (2016). Advances on computational methods for identifying the targets of microRNAs: a review. *Curr. Bioinformatics* 11 1–1. 10.1093/bib/bbw042 27273287

[B11] Li C. Zhao M. Zhang C. Zhang W. Zhao X. Duan X. (2016). miR210 modulates respiratory burst in *Apostichopus japonicus* coelomocytes via targeting Toll-like receptor. *Dev. Comp. Immunol.* 65 377–381. 10.1016/j.dci.2016.08.008 27545641

[B12] Li R. Li Y. Kristiansen K. Wang J. (2008). SOAP: short oligonucleotide alignment program. *Bioinformatics* 24 713–714. 10.1093/bioinformatics/btn025 18227114

[B13] Li S. C. Liao Y. L. Chan W. C. Ho M. R. Tsai K. W. Hu L. Y. (2011). Interrogation of rabbit miRNAs and their isomiRs. *Genomics* 98 453–459. 10.1016/j.ygeno.2011.08.008 21930198

[B14] Liu G. Liu R. Li Q. Tang X. Yu M. Li X. (2013). Identification of microRNAs in wool follicles during anagen, catagen, and telogen phases in Tibetan sheep. *PLoS One* 8:e77801. 10.1371/journal.pone.0077801 24204975 PMC3804049

[B15] Liu X. J. Song L. Liu J. Y. Wang S. C. Tan X. H. Bai X. G. (2013). miR-18b inhibits TGF-beta 1-induced differentiation of hair follicle stem cells into smooth muscle cells by targeting SMAD2. *Biochem. Biophys. Res. Commun.* 438 551–556. 10.1016/j.bbrc.2013.07.090 23916701

[B16] Liu Z. Xiao H. Li H. Zhao Y. Lai S. Yu X. (2012). Identification of conserved and novel microRNAs in cashmere goat skin by deep sequencing. *PLoS One* 7:e50001. 10.1371/journal.pone.0050001 23236360 PMC3517574

[B17] Mardaryev A. N. Ahmed M. I. Vlahov N. V. Fessing M. Y. Gill J. H. Sharov A. A. (2010). Micro-RNA-31 controls hair cycle-associated changes in gene expression programs of the skin and hair follicle. *FASEB J.* 24 3869–3881. 10.1096/fj.10-160663 20522784 PMC4048940

[B18] Miao L. Chen H. Shao Y. Li C. Wei X. Zhang W. (2017). miR-137 modulates coelomocyte apoptosis by targeting 14-3-3ζ in the sea cucumber *Apostichopus japonicus*. *Dev. Comp. Immunol.* 67 86–96. 10.1016/j.dci.2016.11.008 27832949

[B19] Mohammed I. Ahmed A. N. M. Christopher J. Lewis Andrey A. Sharov Natalia V. (2011). MicroRNA-21 is an important downstream component of BMP signalling in epidermal keratinocytes. *J. Cell Sci.* 124 3399–3404. 10.1242/jcs.086710 21984808 PMC3196856

[B20] Nie Y. Li S. Zheng X. T. Chen W. Li X. Liu Z. (2018). Transcriptome reveals long non-coding RNAs and mRNAs involved in primary wool follicle induction in carpet sheep fetal skin. *Front. Physiol.* 9:446. 10.3389/fphys.2018.00446 29867522 PMC5968378

[B21] Paus R. Foitzik K. (2004). In search of the &ldquo hair cycle clock&rdquo: a guided tour. *Differentiation* 72 489–511. 15617561 10.1111/j.1432-0436.2004.07209004.x

[B22] Rendl M. Polak L. Fuchs E. (2008). BMP signaling in dermal papilla cells is required for their hair follicle-inductive properties. *Genes Dev.* 22 543–557. 10.1101/gad.1614408 18281466 PMC2238674

[B23] Ryan D. Oliveira-Fernandes M. Lavker R. (2006). MicroRNAs of the mammalian eye display distinct and overlapping tissue specificity. *Mol. Vis.* 12 1175–1184. 17102797

[B24] Shao Y. Li C. Xu W. Zhang P. Zhang W. Zhao X. (2017). miR-31 links lipid metabolism and cell apoptosis in bacteria-challenged *Apostichopus japonicus* viatargeting CTRP9. *Front. Immunol.* 8:263. 10.3389/fimmu.2017.00263 28348559 PMC5346533

[B25] Wei T. L. Orfanidis K. Xu N. Janson P. Stahle M. Pivarcsi A. (2010). The expression of microRNA-203 during human skin morphogenesis. *Exp. Dermatol.* 19 854–856. 10.1111/j.1600-0625.2010.01118.x 20698882

[B26] Yi R. O’carroll D. Pasolli H. A. Zhang Z. H. Dietrich F. S. Tarakhovsky A. (2006). Morphogenesis in skin is governed by discrete sets of differentially expressed microRNAs. *Nat. Genet.* 38 356–362. 16462742 10.1038/ng1744

[B27] Yi R. Poy M. N. Stoffel M. Fuchs E. (2008). A skin microRNA promotes differentiation by repressing ‘stemness’. *Nature* 452 225–U269. 10.1038/nature06642 18311128 PMC4346711

[B28] Yu J. Ryan D. G. Getsios S. Oliveira-Fernandes M. Fatima A. Lavker R. M. (2008). MicroRNA-184 antagonizes microRNA-205 to maintain SHIP2 levels in epithelia. *Proc. Natl. Acad. Sci. U.S.A.* 105 19300–19305. 10.1073/pnas.0803992105 19033458 PMC2587229

[B29] Yuan C. Wang X. Geng R. He X. Qu L. Chen Y. (2013). Discovery of cashmere goat (Capra hircus) microRNAs in skin and hair follicles by Solexa sequencing. *BMC Genomics* 14:511. 10.1186/1471-2164-14-511 23889850 PMC3765263

[B30] Yue Y. J. Guo T. T. Yuan C. Liu J. B. Guo J. Feng R. L. (2016). Integrated analysis of the roles of long noncoding RNA and coding RNA expression in sheep (*Ovis aries*) Skin during Initiation of Secondary Hair Follicle. *PLoS One* 11:e0156890. 10.1371/journal.pone.0156890 27276011 PMC4898689

[B31] Zhang L. Nie Q. Su Y. Xie X. Luo W. Jia X. (2013). MicroRNA profile analysis on duck feather follicle and skin with high-throughput sequencing technology. *Gene* 519 77–81. 10.1016/j.gene.2013.01.043 23384715

[B32] Zhang L. Stokes N. Polak L. Fuchs E. (2011). Specific microRNAs are preferentially expressed by skin stem cells to balance self-renewal and early lineage commitment. *Cell Stem Cell* 8 294–308. 10.1016/j.stem.2011.01.014 21362569 PMC3086714

[B33] Zhang Y. Tomann P. Andl T. Gallant N. M. Huelsken J. Jerchow B. (2009). Reciprocal requirements for EDA/EDAR/NF-kappaB and Wnt/beta-catenin signaling pathways in hair follicle induction. *Dev. Cell* 17 49–61. 10.1016/j.devcel.2009.05.011 19619491 PMC2859042

[B34] Zhu Z. He J. Jia X. Jiang J. Bai R. Yu X. (2010). MicroRNA-25 functions in regulation of pigmentation by targeting the transcription factor MITF in alpaca (*Lama pacos*) skin melanocytes. *Domest. Anim. Endocrinol.* 38 200–209. 10.1016/j.domaniend.2009.10.004 20036482

